# *Dies1/VISTA* expression loss is a recurrent event in gastric cancer due to epigenetic regulation

**DOI:** 10.1038/srep34860

**Published:** 2016-10-10

**Authors:** Patrícia Oliveira, Joana Carvalho, Sara Rocha, Mafalda Azevedo, Inês Reis, Vânia Camilo, Bárbara Sousa, Sofia Valente, Joana Paredes, Raquel Almeida, David Huntsman, Carla Oliveira

**Affiliations:** 1 i3S - Instituto de Investigação e Inovação em Saúde, Universidade do Porto, Rua Alfredo Allen, 208, 4200-135 Porto, Portugal; 2 IPATIMUP- Institute of Molecular Pathology and Immunology of the University of Porto, Rua Júlio Amaral de Carvalho,45, 4200-465 Porto, Portugal; 3 Dept. Pathology and Oncology, Faculty of Medicine, University of Porto, 4200-465 Porto, Portugal; 4 British Columbia Cancer Agency (BCCA), Vancouver V5Z 4E6, Canada

## Abstract

Dies1/VISTA induces embryonic stem-cell differentiation, via BMP-pathway, but also acts as inflammation regulator and immune-response modulator. Dies1 inhibition in a melanoma-mouse model led to increased tumour-infiltrating T-cells and decreased tumour growth, emphasizing *Dies1* relevance in tumour-microenvironment. Dies1 is involved in cell de/differentiation, inflammation and cancer processes, which mimic those associated with Epithelial-to-Mesenchymal-Transition (EMT). Despite this axis linking *Dies1* with EMT and cancer, its expression, modulation and relevance in these contexts is unknown. To address this, we analysed *Dies1* expression, its regulation by promoter-methylation and *miR-125a-5p* overexpression, and its association with BMP-pathway downstream-effectors, in a TGFβ1-induced EMT-model, cancer cell-lines and primary samples. We detected promoter-methylation as a mechanism controlling *Dies1* expression in our EMT-model and in several cancer cell-lines. We showed that the relationship between *Dies1* expression and BMP-pathway effectors observed in the EMT-model, was not present in all cell-lines, suggesting that *Dies1* has other cell-specific effectors, beyond the BMP-pathway. We further demonstrated that: *Dies1* expression loss is a recurrent event in GC, caused by promoter methylation and/or *miR-125a-5p* overexpression and; GC-microenvironment myofibroblasts overexpress *Dies1.* Our findings highlight *Dies1* as a novel player in GC, with distinct roles within tumour cells and in the tumour-microenvironment.

Dies1/VISTA (ENSG00000107738, ENSMUSG00000020101^1^) is a type-I membrane protein, which induces terminal differentiation of embryonic stem cells (ESCs) into neurons or cardiomyocytes, via the Bone Morphogenetic Protein (BMP)-signalling pathway^2^. This occurs under the control of a regulatory loop involving BMP4 activation of *miR-125a-5p*, which in turn is capable of directly repressing Dies1 transcription, halting ESC differentiation^3^. Dies1 also plays a crucial regulatory role during adipocyte differentiation, however independently of BMP-signalling. In particular, upregulation of Dies1 was demonstrated exclusively in differentiated fat cells and its knockdown inhibited adipocyte differentiation^4^. Dies1 has also been implicated in inflammation, due to the demonstration of chronic multi-organ inflammation and increased inflammatory chemokines’ serum levels in Dies1-knockout mice^5^. Although this mouse model lacked systemic or organ-specific autoimmune disease, an increased population of activated peripheral T-cells was observed, in agreement with previous data showing that antigen presenting cells (APCs) expressing Dies1/VISTA are able to directly suppress T-cell proliferation^6^. Similar observations were reported by Yoon *et al*., which have also shown that Dies1 (DD1α) works as a homophilic receptor in T-cells, as well as a key molecule interacting simultaneously with apoptotic cells and macrophages, facilitating phagocytic engulfment. Interestingly, this process was shown to be tightly controlled by p53, as Dies1 was shown to be a downstream target of this tumour suppressor gene^7^. Supporting the role of *Dies1* as an immune-response regulator, Le Mercier *et al*. have also shown that Dies1 blockade, using a monoclonal antibody, caused an increase in tumour-infiltrating T-cells and decreased tumour growth, in an inducible melanoma transgenic mouse model^6^. Altogether, the described roles credited to Dies1 span the fields of differentiation, inflammation and cancer.

Epithelial to mesenchymal transition (EMT) occurs naturally during embryogenesis, but may also occur associated with pathological contexts of chronic inflammation, fibrosis and cancer^8^,^9^,^10^. This process increases cellular plasticity, through a large scale cellular reprogramming, that promotes a shift from differentiated, polarized epithelial cells to dedifferentiated mesenchymal-like cells^8^,^9^,^10^,^11^. EMT is thought to bestow cancer cells with *de novo* migratory and invasion properties, enabling cancer progression and distant organ colonization. In addition, EMT has been shown to generate cells with increased stem-like properties, either by enrichment in stem cell subpopulations and/or increased expression of stem cell markers, such as CD44 and CD24^11^,^12^,^13^. EMT can be triggered in epithelial cells, by inflammatory mediators such as TGFβ1, TNFα and interleukins thought to be produced by immune cells from the microenvironment. As a consequence, EMT inducers like Snail and Twist become overexpressed and downregulate the EMT hallmark protein E-cadherin^14^. These molecules are all important players in cancer with impact in cancer progression and patient´s prognosis^9^,^15^,^16^,^17^. EMT is therefore a process also involved in differentiation, inflammation and cancer.

Increased exposure of epithelial cells to inflammatory factors during chronic inflammation can favour tumourigenesis^18^,^19^. This association between inflammation and tumourigenesis may provide a mechanistic explanation for the augmented cancer risk in some inflammatory disorders. This is particularly valid for the gastrointestinal tract, which is highly exposed to pro-inflammatory factors produced by the gut microbiome, presenting a persistent chronic low-level inflammation state^20^. Upon oncogenic transformation, epithelial cells exposed to inflammatory signals may be more resistant to elimination by the immune system, which in turn promotes tumour progression^20^. Several studies have shown that areas of gastric adenocarcinoma frequently overlap with regions of chronic inflammation. In fact, upon chronic inflammation, parietal cells and chief cells in the gastric mucosa may be lost, leading to a reduction in signals for growth and differentiation of gastric progenitors^21^. Transgenic mice with specific ablation of parietal cells, revealed an accumulation of undifferentiated progenitors with increased levels of proliferation and occurrence of intestinal metaplasia, a crucial step in Correa’s pathway for gastric adenocarcinoma development^22^. During the process of gastric tumourigenesis, signalling pathways such as the PI3K/AKT, Wnt/β-catenin, Notch and TGF-β become activated and may trigger cancer-related EMT, either transiently or persistently^23^,^24^,^25^,^26^. Loss of *CDH1*/E-cadherin expression and increased cancer stem cell self-renewal are processes that often take place when gastric cancer (GC) is developing^26^. GC is not the single example of inflammation-associated cancer, as colorectal cancer risk also increases with the severity of inflammation in the setting of ulcerative colitis^27^. In this nonspecific inflammatory condition, characterized by alternating periods of active disease and remission, it has been shown consistent up-regulation of an EMT-related signature, specifically in the intestinal mucosa of active ulcerative colitis patients’^27^. Altogether these examples support the existence of an axis involving cellular differentiation, chronic inflammation, EMT and tumour progression. Interestingly, this axis appears to overlap the diverse roles of Dies1 as differentiation inducer, inflammation regulator and cancer immunity modulator. In the present study, we used a TGFβ1-induced EMT model, epithelial cancer cell lines and cancer samples to dissect the mechanisms underlying *Dies1* expression and disclose its role in epithelial carcinogenesis.

## Results

In the present study, we analysed the expression and regulation of *Dies1* in a TGFβ1-induced EMT model, epithelial cancer cell line models and cancer samples, aiming at disclosing a role for this molecule in epithelial carcinogenesis, and the mechanisms that may control its expression and signalling.

### *Dies1* expression and its promoter methylation status vary along EMT and MET

We started by assessing *Dies1* mRNA expression variation in an EMT/MET *in vitro* model, that we established using the spontaneously immortalized normal mammary epithelial cell line, EpH4^28^. By subjecting the parental EpH4 cells (E-cells) to a 7-day treatment with the inflammatory cytokine TGFβ1, we obtained a culture of mesenchymal cells (M-cells), with a classical fibroblastoid morphology, downregulation or functional inactivation of several epithelial markers (for example, *Ocln* and *Mgat3* downregulation, and E-cadherin deregulation^28^), and upregulation of mesenchymal markers (i.e. *Vim*, *Zeb2*, *Twist1*). Upon TGFβ1 removal from the culture medium, M-cells re-acquired a cobblestone morphology, creating a Reversed-Epithelial cell population (RE-cells). Overall, RE-cells displayed an expression pattern for epithelial and mesenchymal markers similar to E-cells, with a recovery of an epithelial-like signature^28^. Our EMT/MET *in vitro* model simulates the dynamics observed during reversible inflammation-induced cellular dedifferentiation, with M-cells constituting a less differentiated population in opposition to the more differentiated E and RE-cells.

**Figure 1:**
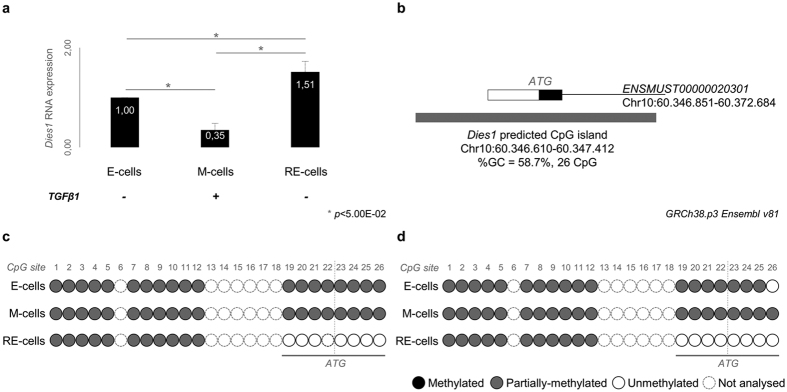
*Dies1* expression and regulation across an *in vitro* model of EMT and MET. (**a)** *Dies1* expression in E-, M- and RE-cells. Asterisks stand for significantly distinct comparisons (*p* < 5.00E-02). (**b)** Schematic representation of *Dies1* predicted CpG island in mouse. White rectangle for *Dies1* 5′UTR in exon 1. Black rectangle for coding region of exon 1. Grey rectangle for the predicted CpG island. (**c**,**d**) Two examples of the results obtained for the direct-sequencing of *Dies1* predicted CpG island. Representation of each CpG site using white circles for unmethylated CpG sites, grey circles for partially-methylated CpG sites and black circles for fully methylated CpG sites.

By qRT-PCR we observed that *Dies1* expression became significantly downregulated in M-cells when compared to E-cells (*p* = 1.00E-03, Fig. 1a). Interestingly, in RE-cells *Dies1* expression was recovered to levels even higher than in the original E-cells (*p* = 1.30E-03 for M *vs.* RE-cells, *p* = 1.40E-02 for E *vs.* RE-cells, Fig. 1a). We next investigated whether this alteration in *Dies1* RNA expression could be caused by alterations in the methylation status of its gene promoter. Using Ensembl database^1^ and the web tool “CpG Island Searcher” 29, we detected a possible CpG island encompassing 26 CpG sites at the 5′-end of *Dies1* locus (GRCm38.p4, Chr10:60.346.610-60.347.412, %GC  =  58.7%, Fig. 1b). Sequencing of E, M and RE-cells bisulfite-treated DNA revealed that at least 7/8 CpG sites were recurrently methylated in E and M cells, and demethylated in RE-cells (CpG sites 19-26, examples of 2 biological replicates in Fig. 1c and Supplementary Fig. S1). Importantly, these CpG sites overlap with the start codon of *Dies1* (ATG at chr10: 60.347.022 for ENSMUST00000020301)^1^. The fluctuation in *Dies1* expression in RE-cells could therefore be associated with the methylation pattern at these specific CpG sites: E and M-cells presenting lower expression and denser methylation, while RE-cells presenting increased *Dies1* expression and some degree of demethylation.

### *Dies1* modulates the expression of its downstream effectors *ID2* and *ID3* in an EMT/MET model

**Figure 2:**
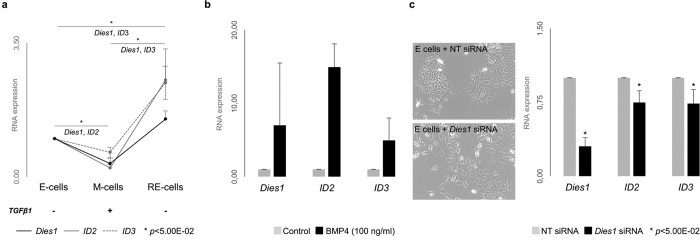
*Dies1* and BMP-pathway effectors expression in E-, M-, RE- cells and in E-cells with specific inhibition of *Dies1*. (**a)** Expression of *Dies1*, *ID2* and *ID3* in E-, M- and RE-cells. Asterisks stand for significantly distinct comparisons (*p* < 5.00E-02). (**b)** Expression of *Dies1*, *ID2* and *ID3* in E-cells after exogenous stimulation with BMP4 (*Dies1* expression fold change from 2.0x to 16.8x, *ID2* expression fold change from 11.3x to 17.2x, and *ID3* expression fold change from 1.5x to 7,1x). (**c)** Brightfield images of E-cells transfected with Non-Targeting siRNA (NT siRNA) and *Dies1* siRNA and expression of *Dies1*, *ID2* and *ID3*.

Previous studies have shown that Dies1 is a crucial modulator of stem cell differentiation, exerting its regulatory action in either a BMP-pathway dependent or independent manner^2^,^4^. To understand whether Dies1 effects were BMP-signalling dependent in our EMT/MET model, we assessed the expression of *ID2* and *ID3*, which have been previously described as downstream effectors of Dies1 and BMP^2^. By qRT-PCR, we verified that *ID2* and *ID3* closely followed the expression pattern of *Dies1* across EMT/MET (Fig. 2a). *ID2* and *ID3* were both downregulated in M-cells (*ID2* statistically significant*: p* = 5.91E-03 for E *vs.* M-cells) and upregulated in RE-cells (*ID3* statistically significant: *p* = 2.98E-02 for M vs. RE-cells and 4.15E-02 for E vs. RE-cells). To prove that Dies1 was under the control of the BMP-pathway, we submitted E-cells to a 48h-treatment with exogenous BMP4. In fact, we observed an overall increase in *Dies1* expression (Fig. 2b) as well as of *ID2* and *ID3*, demonstrating that Dies1 is modulated by BMP signalling in E-cells. Next, to understand whether the fluctuation of *ID2* and *ID3* expression was induced by *Dies1* expression changes, we specifically inhibited *Dies1* expression in E-cells by RNAi. Upon 70% inhibition of *Dies1* expression, both *ID2* and *ID3* were significantly downregulated (NT siRNA vs. *Dies1* siRNA: *p* = 5.66E-03 for *ID2* and *p* = 3.51E-03 for *ID3*, Fig. 2c). These results demonstrate that *ID2* and *ID3* expression is modulated by Dies1 in E-cells.

### The expression of *Dies1* and its downstream targets is generally downregulated in epithelial cancer cell lines and may be explained by epigenetic mechanisms

**Figure 3:**
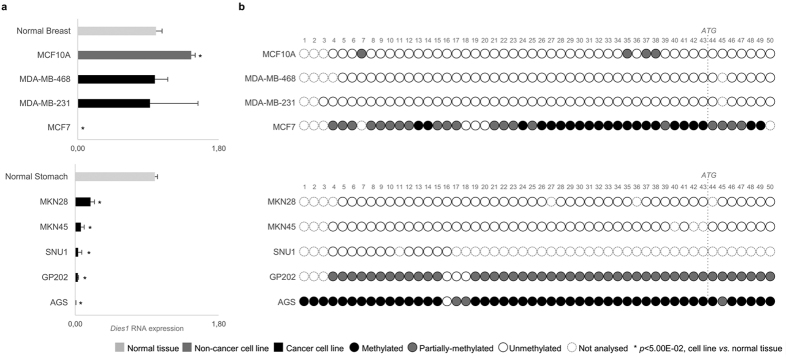
*Dies1* expression and promoter methylation status in a panel of breast cancer, gastric cancer and colorectal cancer cell lines. (**a)** *Dies1* expression in a panel of breast cancer cell lines (MDA-MB-468, MDA-MB-231 and MCF7) normalized to normal breast tissue and of gastric cancer cell lines (MKN28, MKN45, SNU1, GP202 and AGS) normalized to normal gastric tissue. Also included is the near-normal breast cell line MCF10A (non-cancer, dark grey bar). Light grey bars to normal tissues and black bars correspond to cancer cell lines. Asterisks stand for significantly distinct comparisons (*p* < 5.00E-02). (**b)** *Dies1* promoter methylation status in all described cancer cell lines (and MCF10A). Representation of each CpG site using white circles for unmethylated CpG sites, grey circles for partially-methylated CpG sites, black circles for fully methylated CpG sites and dashed white circles for unassessed CpG sites.

Given the observed variation of *Dies1* expression during EMT/MET and the increasing relevance of these processes for cancer progression^13^,^23^,^30^, we evaluated the expression of *Dies1* in 12 cell lines derived from epithelial cancer: 3 from breast, 5 from gastric and 4 from colon. We also analysed normal breast, gastric and colon tissues and an immortalized normal breast epithelial cell line (MCF10A) (Fig. 3a, Supplementary Fig. S2). *Dies1* was found significantly downregulated in 9/13 cancer cell lines in comparison with corresponding normal tissues and/or normal cancer cell line: 1/4 breast; 3/4 colon; and 5/5 gastric cancer cell lines (Fig. 3a, Supplementary Fig. S2). Importantly, all GC cell lines displayed consistently very low or no expression of *Dies1* in comparison with normal stomach (*p* < 1.16E-03, Fig. 3a).

Given that *Dies1* expression could be modulated by promoter methylation, we next investigated the methylation status of *Dies1* promoter in all cancer cell lines. Using the same approach described for the murine *Dies1* locus, we were able to identify a *CpG* island upstream of the human *Dies1* locus, encompassing 50 CpG sites (*CpG* island located at chr10:71.773.335-71.773.735, GRCh38.p3 Ensembl v81, Fig. 3b, Supplementary Fig. S2). The breast cancer cell line MCF7 displayed low *Dies1* expression levels and concomitant widespread methylation across *Dies1* promoter. In contrast, MCF10A, MDA-MB-468 and MDA-MB-231 displayed *Dies1* expression at the same levels of normal breast tissue, and an almost completely demethylated *Dies1* promoter. These data demonstrated a direct relationship between *Dies1* expression and the methylation status of its promoter in all breast cell lines analysed (Fig. 3b). The promoter methylation analysis recurrently failed for colon cancer cell lines, with a single cell line (RKO) displaying 9 methylated CpG sites out of the 12 that we succeeded to analyse (Supplementary Fig. S2). In GC cell lines, the overall downregulation of *Dies1* expression was followed by extensive promoter methylation in two cell lines (AGS and GP202), while in the other two (MKN45 and MKN28) the gene promoter remained demethylated, despite the very low *Dies1* expression levels (Fig. 3b). Overall, these data suggest that *Dies1* expression is downregulated in epithelial cancer derived cell lines, and that as in the EMT/MET model, *Dies1* expression can be controlled by methylation at the gene promoter in a fraction of the cell lines analysed.

**Figure 4:**
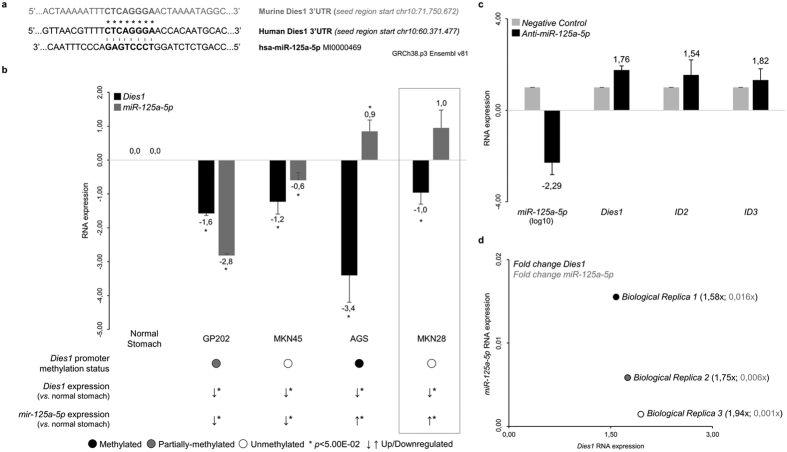
*Dies1* expression and *miR-125a-5p* in gastric cancer cell lines. (**a)** Conservation of the murine and human *Dies1* seed region for miR-125a-5p. Asterisks stand for conserved nucleotides, dashes for complementary regions. (**b)** *Dies1* and *miR-125a-5p* expression in gastric cancer cell lines in comparison with normal stomach mucosa (in log scale). Also included is a summary of the Dies1 promoter methylation status (same legend as in Fig. 1 applies), for comparison and correlation purposes. Asterisks stand for significantly distinct comparisons (*p* < 5.00E-02). (**c)** RNA expression analysis confirming miR-125a-5p inhibition (from 0,016x to 0,001x *vs.* negative control) and concomitance increased expression of Dies1 (from 1,58x to 1,94x *vs.* negative control), ID2 (from 0,87x to 2,19x *vs.* negative control) and ID3 (from 0,92x to 1,87x *vs.* negative control. (**d)** Representation of the RNA expression analysis results correlating miR-125a-5p expression with Dies1 expression for each biological replicate performed.

Given the massive *Dies1* downregulation in GC cell lines, and in order to identify a potential mechanism that could downregulate *Dies1* in the two GC cell lines (MKN45 and MKN28) lacking promoter methylation, we explored the expression of *miR-125a-5p*, known to target *Dies1* 3′-UTR in mouse ESCs^31^, in these two GC cell lines. We first confirmed that the murine *Dies1* 3′-UTR target region for *miR-125a-5p* was perfectly conserved with a region within the human *Dies1* 3′UTR (starting at chr10: 60.371.477, Fig. 4a). Next, we used the web tool MiRWalk 2.0^32^ to predict all possible miRNAs targeting the human *Dies1* 3′UTR. We observed that all five selected databases, included within MiRWalk, consistently predicted a binding site for *miR-125a-5p* in the same region of the human *Dies1* 3′-UTR, which was seen to be conserved with the murine *Dies1* 3′-UTR target region for *miR-125a-5p*. We therefore analysed the expression of *miR-125a-5p*, by qRT-PCR, in four GC cell lines: GP202 and AGS with widespread *Dies1* promoter methylation and; MKN45 and MKN28 without *Dies1* promoter methylation. We observed that in GP202 and MKN45, *miR-125a-5p* was significantly downregulated in comparison with normal stomach (*p* < 2.32E-04, Fig. 4b), hence excluding it as responsible for *Dies1* downregulation. In AGS and MKN28, *miR-125a-5p* was found to be overexpressed (*p* = 4.47E-02 for AGS and *p* > 5.00E-02 for MKN28) in comparison with normal stomach (Fig. 4b). These observations suggest that in AGS, promoter methylation and *miR-125a-5p* overexpression could be acting in concomitance, while in MKN28, *Dies1* downregulation could be solely due to *miR-125a-5p* overexpression. To understand whether miR-125a-5p was directly targeting Dies1, we next specifically inhibited miR-125a-5p expression in MKN28 cells, using antimiRs technology.Three biological replicates were performed, all successfully inhibiting *miR-125a-5p* expression with concomitant increase in *Dies1* expression (Fig. 4c). Of notice, *Dies1* expression was found to be strongly correlated with the efficiency of *miR-125a-5p* inhibition (Fig. 4d, R^2^ = 0,94). Concerning *ID2* and *ID3*, an overall trend for increased expression was observed upon *miR-125a-5p* downregulation (Fig. 4c). These results reinforce that in MKN28, *Dies1* expression is under the control of *miR-125a-5p*. Since, it has been recently described that *Dies1* is a downstream target of p53, we tried to understand whether mutations in *TP53* could also constitute a mechanism underlying *Dies1* downregulation in GC cells lines. Indeed, COSMIC data showed that MKN45 carries a TP53 mutation (heterozygous R110C^33^) that may explain *Dies1* downregulation. Additionally, MKN28 also carried a TP53 mutation (homozygous I251L^33^) besides *miR-125a-5p* upregulation.

We also assessed *ID2* and *ID3* mRNA expression across the same panel of human cell lines (*n* = 13, Supplementary Fig. S2). Unlike what was observed for *Dies1* in breast cancer cell lines, *ID2* and *ID3* were significantly downregulated in all cell lines (*p* < 2.72E-03), demonstrating no relationship between *Dies1* and its potential downstream targets *ID2* and *ID3* in this cancer type. In contrast, 3/4 colon cancer cell lines exhibited *ID2* or *ID3* expression loss concomitantly with *Dies1* downregulation (Supplementary Fig. S2). In 5/5 GC cell lines, *ID2* or *ID3* expression was low or undetectable in comparison with normal stomach (*p* < 7.67E-03), and in agreement with *Dies1* downregulation. In summary, *Dies1* expression downregulation seems particularly relevant in GC, as this is a consistent feature of all GC cell lines analysed. This downregulation could be explained *via Dies1* promoter methylation, miR-125a-5p overexpression, or a combination of both, and even through the presence of mutant p53. Moreover, as *ID2* and *ID3* follow the *Dies1* expression trend in GC, it is likely that they act as*Dies1* downstream targets also in the GC model.

### Expression of *Dies1* and *ID3* is recurrently downregulated in primary GC samples

**Figure 5:**
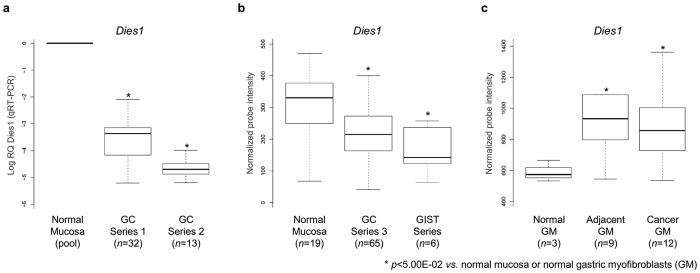
*Dies1* expression in three independent GC Series, six GIST samples and in a series of normal, adjacent and cancer-associated gastric myofibroblasts (GM). (**a)** *Dies1* expression in GC Series 1 and 2 normalized to a pool of normal gastric mucosa. Asterisks stand for significantly distinct comparisons (*p* < 5.00E-02). (**b)** *Dies1* expression in a pool of normal mucosas, in GC Series 3 and in six GIST samples. (**c**) Dies1 expression in a series of normal, adjacent and cancer-associated gastric myofibroblasts.

The consistent downregulation of *Dies1* in GC cell lines led us to investigate the relevance of its expression in primary GC samples. We assessed, by qRT-PCR, the expression of *Dies1* in 45 samples from two independent GC series available to us in light of ongoing collaborations with São João Hospital (GC series 1: 32 samples; GC series 2: 13 samples). *Dies1* was significantly downregulated in both GC series in comparison to a pool of normal stomach mucosas (GC series 1: *p* = 5.00E-03; GC series 2: *p* = 1.00E-02 for GC, Fig. 5a). To re-enforce these results, we also mined the gene expression profiles of GC samples assessed by microarray technology for which data was deposited and available for analysis at the ArrayExpress database (E-GEOD-26942^34^). This experiment encompassed 65 GC human samples (GC series 3), 6 gastrointestinal stromal tumour (GIST series) samples, and 19 normal stomach mucosa samples, used for comparison purposes. Mimicking the results obtained for GC series 1 and 2, *Dies1* expression was significantly downregulated in samples from GC series 3 and GIST samples, in comparison with normal mucosas (GC series 3: *p* = 1.60E-03; GIST: *p* = 3.40E-03, Fig. 5b). Next, we tried to understand whether *Dies1* downregulation was also detected at the protein level, using three anti-Dies1 antibodies. These antibodies were tested for immunohistochemistry, immunocytochemistry and Western Blot, using normal stomach mucosa samples, as well as human cell lines. However, inconsistent and non-reproducible results were obtained, *e.g.* different staining patterns within the same histological structure (Supplementary Fig. S3). Therefore, Dies1 protein expression assessment was not further pursued.

Following the same rational used for cell lines, we were able to assess the *TP53* status (WT or mutated) in the 32 GC samples encompassing Series 1. This information was available as these 32 GC samples have been subjected to Whole Genome Sequencing in a parallel ongoing project of the group. We found that 9/32 (28.1%) of GC samples displayed somatic *TP53* mutations: frameshift mutation TP53_g.14066G > A; missense mutations TP53_g.12521A > C, TP53_g.13794G > A, TP53_g.13370G > A, TP53_g.13379C > T, TP53_g.13824C > T, TP53_g.13797C > T or TP53_g.13779C > T and; nonsense mutation TP53_g.12706C > T. We further assessed *ID2* and *ID3* expression in GC series 3 and in the GIST series. We detected a significant downregulation of *ID3* in GC series 3 in comparison with the normal stomach mucosa samples (*p* = 1.42E-05), but *ID2* expression remained unchanged in this experiment (Supplementary Fig. S4).

In summary, we observed a consistent downregulation of *Dies1* in three independent GC series in comparison with normal gastric mucosa that was mimicked by *ID3* expression downregulation.

### Expression of *Dies1* is increased in myofibroblasts from the GC microenvironment

Recent findings have shown that *Dies1* is overexpressed in APCs within the tumour microenvironment, directly suppressing T-cell proliferation^6^. To understand whether *Dies1* expression could be detected in other tumour microenvironment components, we analysed data derived from a set of gene expression microarrays experiments deposited in the ArrayExpress database (E-GEOD-44740)^34^, from Balabanova *et al*.^35^. We assessed *Dies1* expression in primary cultures of myofibroblasts isolated from: gastric tumours (*n* = 12); normal tissue adjacent to gastric tumours (*n* = 9), and; normal tissue from healthy donors (*n* = 3). We observed that in both adjacent and cancer-associated gastric myofibroblasts, *Dies1* expression was significantly increased in comparison with gastric tissue myofibroblasts from healthy donors (*p* = 3.60E-02 and *p* = 3.10E-02, respectively, Fig. 5c). Concerning *ID2* and *ID3* expression, no significant alterations were detected among the three types of gastric myofibroblasts (Supplementary Fig. S4). Overall, in cancer-associated or cancer-adjacent gastric myofibroblasts *Dies1* is overexpressed, however the expression of its potential downstream targets, *ID2* and *ID3*, does not follow the same trend. These data support distinct roles for *Dies1* in tumour cells and in the surrounding microenvironment, and suggest that *Dies1* may signal through distinct pathways, depending on the cell type.

## Discussion

The aim of this study was to explore the relevance of *Dies1* gene expression, and its potential downstream targets, in an *in vitro* model mimicking TGFβ1-associated inflammation and cellular transdifferentiation, and in epithelial cancer. We started by assessing *Dies1* expression and its promoter methylation status in a TGFβ1-induced EMT *in vitro* model. This model was designed to recreate a TGFβ1-induced inflammation process known to be relevant for both cellular differentiation and cancer. We verified that epithelial cells treated with TGFβ1 to trigger EMT, significantly decrease *Dies1* expression without any promoter methylation changes. However, when cells recovered from EMT regaining an epithelial-like phenotype, *Dies1* became overexpressed and specific promoter CpG sites became demethylated. These results are particularly interesting when considering that EMT is a process that can create cells with increased self-renewal ability and multi-lineage differentiation potential^36^. Following on this idea, it is valid to assume that cells that underwent EMT became dedifferentiated in comparison with the original epithelial population (*Dies1* expression decreased), and that upon TGFβ1 removal, cells differentiated towards an epithelial phenotype (*Dies1* expression increased). It seems therefore that in the present model, and as previously described in ESCs^2^, *Dies1* expression is necessary for the reacquisition of a more differentiated phenotype, and may act as a regulator of cellular differentiation. We also show for the first time that *Dies1* expression is controlled by promoter methylation.

Following the data described for ESCs^2^, in our EMT/MET model the expression variation of BMP-signalling effectors *ID2* and *ID3* closely mimicked that of *Dies1*, supporting *Dies1* role as a differentiation regulator acting in a BMP-dependent manner. Aloia *et al*. had shown that the *Dies1* role in ESCs differentiation was associated and dependent to BMP-signalling: on one hand, upon *Dies1* knockdown, *ID1*/*2*/*3* expression was lost and ESCs were not allowed to differentiate; on the other hand, when combining *Dies1* knockdown with chemical suppression of the BMP-signalling receptor *Alk3*, ESCs overcame *Dies1* absence and became more differentiated. To understand whether this crosstalk between *Dies1* and BMP-signalling was also valid in our model, we first stimulated this pathway in our E-cells with exogenous BMP4 treatment and observed an increase in *Dies1* expression, as well as *ID2*/*ID3.* This observation proved that *Dies1* was under the control of the BMP-pathway in our model. Next, we specifically inhibited *Dies1* expression in E-cells, as *Dies1* expression was too low in M-cells to allow further downregulation. *Dies1* depletion led to a decrease in *ID2*/*ID3* expression, in agreement with Aloia *et al*. data, supporting a bidirectional relationship between *Dies1* and BMP-signalling in our model. Also of notice was the fact that *BMP4* expression was found to be specifically downregulated in M-cells, which could explain the expression downregulation of *Dies1* in these cells (Supplementary Fig. S1). In fact, M-cells are generated by exogenous treatment with TGF-β1, which might be diverting the shared cofactor Smad4 signaling from the BMP-pathway (likely active in E-cells) towards the TGFβ-pathway. Nevertheless, further experiments ought to be performed to confirm this hypothesis. Taken together, our results suggest a role for *Dies1* in TGFβ1-induced EMT/MET, with increased *Dies1* expression associated with more differentiated cellular states (E- and RE-cells).

In light of the above results and literature data associating Dies1 with cancer, we assessed *Dies1* expression and its promoter methylation status across a panel of epithelial cancer cell lines. We observed a complete correlation between *Dies1* expression and promoter methylation status in breast cancer cell lines, as those that were demethylated expressed *Dies1* and those that were methylated did not. To the best of our knowledge, this is the first epigenetic mechanism leading to *Dies1* downregulation described in breast cancer cell lines. We excluded an exclusive relationship between *Dies1* and BMP-signalling in the breast cancer model by assessing the expression of *ID2*/*ID3,* as these genes were systematically downregulated independently of *Dies1* expression. In addition, one of the breast cancer cell lines used, MDA-MB-468, has an expression-impairing deletion in the Smad4 gene, hence decoupling *Dies1* expression from BMP-signalling^37^. The observations in the breast cancer cell lines are in line with findings by Ren *et al*.^4^ who showed that *Dies1* silencing inhibited adipogenesis without altering the levels of BMP-pathway regulators such as phospho-Smad1, even in the presence of the BMP-receptor ligand, BMP4^4^. Altogether, these data mainly demonstrate the limited role for Dies1 in breast cancer and highlight the fact that the BMP-pathway and the expression of its targets is independent from Dies1.

In the colon model, although *ID2*/*ID3* expression paralleled *Dies1* expression in all cell lines analysed, 2/3 cell lines are Smad4-deficient^38^,^39^ and therefore irresponsive to the BMP-signalling. This suggests that, as seen for the breast cancer cell lines, *Dies1* expression is independent from BMP-signalling in colon cancer.

In the present study, the most consistent cancer-associated data was obtained for the GC model. We have shown that *Dies1* is downregulated in all GC cell lines assessed, regardless of their differentiation status (poorly differentiated: AGS, SNU1, MKN45, GP202; moderately differentiated: MKN28) or their EMT signature (metastable: MKN45, MKN28, AGS; mesenchymal-like: SNU1)^40^,^41^,^42^,^43^,^44^,^45^. We were able to demonstrate that *Dies1* promoter methylation could control *Dies1* expression in some cell lines, while *miR-125a-5p* expression, a known transcription repressor of *Dies1* in ESCs^31^, can control *Dies1* expression in others. In fact, we proved that in MKN28 cells, which displayed no methylation at the promoter level, *Dies1* expression is under the control of *miR-125a-5p*, as its targeted inhibition led to an overexpression of *Dies1*. Synergistic effects may also be underlying *Dies1* expression downregulation in GC cell lines: for example, AGS cells displayed the lowest levels of *Dies1*, likely due to the co-occurrence of promoter methylation and *miR-125a-5p* overexpression. Other mechanisms may also help explaining *Dies1* downregulation, such as p53 mutation status, in light of Yoon *et al*. study. For example, MKN45 cells, for which neither *miR-125a-5p* overexpression nor *Dies1* promoter methylation was detected, encode a p53 heterozygous mutation (R110C^33^), which could explain *Dies1* downregulation. In fact, p53 mutations were also detected in MKN28 (homozygous I251L^33^), strengthening the existence of combinatory mechanisms underlying *Dies1* massive downregulation. Nevertheless, p53 mutation status *per se* may not predict *Dies1* expression, given that breast cancer cells MDA-MB-468 and MDA-MB-231, display high levels of *Dies1* and mutations in p53 (homozygous R273H and heterozygous R280K^33^, respectively). Beyond the described genetic/epigenetic mechanisms, we may not exclude a contribution of BMP signalling to the regulation of *Dies1* expression, as we have observed that *Dies1*, *ID2* and *ID3* were concomitantly decreased in all GC cells lines.

The GC cell line data was reinforced by data obtained from three different series of primary GC samples. Strikingly, *Dies1* was significantly downregulated in all GC samples analysed (*n* = 110 samples), in comparison to normal stomach mucosa. We tried to understand whether *Dies1* mRNA downregulation was reflected in Dies1 protein expression loss, using several antibodies and techniques. However, inconsistent results were obtained, showing that further studies should be performed, using other available antibodies, to address whether Dies1 protein expression is also lost in GC.

Interestingly, 9 out of the 32 GC samples from Series 1 displayed somatic *TP53* mutations, which may help explaining *Dies1* downregulation. No information was available concerning *TP53* status in Series 2. *Dies1* was also downregulated in a small series of gastrointestinal stromal tumours (GIST), a fact not surprising given the mesenchymal origin of these tumours^46^, which recalled *Dies1* downregulation in our epithelial cells that undergo EMT. Of notice is the fact that *Dies1* downregulation was followed by *ID3* downregulation, strengthening the association between *Dies1* and BMP-signalling in GC.

Following on the observations that *Dies1* is an immune-response modulator when expressed in cells from the tumour microenvironment^6^,^7^,^47^,^48^, we found that *Dies1* expression was significantly upregulated in cancer-adjacent and cancer-associated gastric myofibroblasts, in comparison with normal gastric myofibroblasts. In fact, the secretome of cancer-associated gastric myofibroblasts has been shown to impact tumour growth, reinforcing the relevance of stromal cells in the tumour microenvironment^49^. Moreover, when overexpressed in APCs from the tumour microenvironment, *Dies1* actively suppressed T-cell proliferation, both *in vitro* and *in vivo,* leading to a decreased anti-tumoral immune response and promoting tumour growth in a T-cell dependent manner^47^. Our observations suggest that *Dies1* may also have a role in other tumour microenvironment components, such as cancer-associated myofibroblasts. In summary, we disclosed promoter methylation as an epigenetic mechanism controlling *Dies1* expression in an inflammation-induced EMT model and in several epithelial cancer cell lines. We have also shown that the relationship between *Dies1* expression and BMP-signalling, found for the EMT model, may be mimicked or not in different cancer types. This suggests that *Dies1* may have other cell-specific effectors, beyond the BMP-pathway. Our results demonstrated that *Dies1* loss of expression is a recurrent event in GC, in concomitance with promoter methylation and in association, or not, with *miR-125a-5p* overexpression, and even *TP53* mutations. Finally, we have also shown that in GC, *ID3* is likely a downstream effector of *Dies1*, and that GC myofibroblasts overexpress *Dies1*, which may be acting together with immune cells to refrain the anti-tumoral immune response, enabling tumour growth.

## Methods

### Cell culture

*EpH4* cell line (E-cells) was cultured in D-MEM/F12-*Glutamax*™ (Invitrogen) supplemented with fetal bovine serum (5%, Lonza), penicillin-streptomycin (1%, Invitrogen) and insulin (5 μg/ml, Sigma-Aldrich). EpH4 cell line was kindly provided by Dr. Angela Burleigh and Dr. Calvin Roskelley from British Columbia Cancer Agency (Vancouver, Canada). Mesenchymal cell cultures (M-cells) were obtained by adding transforming-growth-factor-β1 (TGFβ1, Sigma-Aldrich) during 7 days. Reverted-epithelial cell cultures (RE-cells) were obtained by replacing TGFβ1-enriched medium by normal culture medium for another 4 days. Cell lines MCF10A, MDA-MB-468, MDA-MB-231, MCF7, MKN28, SNU1, MKN45, AGS, SW480, HCT116, HT29 and RKO were acquired from ATCC and cultured using recommended mediums (RPMI/DMEM, Invitrogen, 10% fetal bovine serum Lonza and 1% penicillin-streptomycin, Invitrogen). GP202 was obtained from Ipatimup’s Cell Line Bank^40^ and cultured using recommended mediums (RPMI, Invitrogen, 10% fetal bovine serum Lonza and 1% penicillin-streptomycin, Invitrogen). All cell lines authentication was performed at the Ipatimup’s Cell Lines Bank, using STR amplification (Promega-Powerplex16, Identifiler).

### RNA extraction and expression quantification

RNA was extracted using mirVana miRNA Isolation Kit (Invitrogen) following manufacturer’s instructions from: (1) 3 biological replicas of *EpH4* cell line stages (E, M, RE-cells); (2) all described cell lines and; (3) randomly selected tissue specimens from three GC series (GC Series 1, *n* = 32 GC samples, GC Series 2, *n* = 13 GC samples and *n* = 10 normal gastric mucosa from an available IPATIMUP/Hospital São João dataset, informed consent obtained from all patients, study approved by Hospital S. João Ethics Committee). Of notice, for Series 1 and Series 2, GC cases were reviewed by an expert pathologist and RNA was extracted from microdissected gastric tumours in regions displaying more than 70% of gastric cancer cells. RNA from pools of normal stomach, colon and breast tissue was acquired from Stratagene (Agilent Technologies). RNA was reversed-transcribed using Superscript-II-Reverse-Transcriptase and random-hexamers (Invitrogen). Quantitative-Real-Time-PCR (qRT-PCR) was performed using RNA from 3 biological replicas (E, M, RE-cells and cancer cell lines described), in a ABI Prism 7000 Sequence Detection System and using TaqMan Gene Expression Assays (Applied Biosystems) or PrimeTime qPCR Assays (Integrated DNA Technologies), for the following genes: mouse *CDH1* (Mm00486909_g1), *Vim* (Mm01333430_m1), *ID2* (Mm00711781_m1), *ID3* (Mm00492575_m1), *Dies1* (Mm00472312_m1) and; human *Dies1* (Hs00735289_m1), *ID2* (Hs00171409_m1), *ID3* (Hs00747379_m1) and *miR-125a-5p* (hsa-miR-125a-5p). The endogenous controls used were mouse and human *GAPDH* (Mm99999915_g1, Hs02758991_g1) for cell lines and human *18S* for GC series samples (Hs99999901_s1). All data was analysed by the comparative 2^(−ΔΔCT)^ method^50^ and two-tailed Student’s T-test or Wilcoxon signed-rank test applied^51^.

### DNA extraction and methylation analysis

DNA from 3 independent biological replicas of E, M, RE-cells and described human cancer cell lines was extracted using Invisorb Spin Tissue Mini Kit, following manufacturer’s instructions (STRATEC Molecular). DNA was subjected to bisulfite-conversion, using Epitect Bisulfite Kit, following manufacturer’s instructions (Qiagen). Murine/human *Dies1* promoter methylation analysis was carried out within the *CpG* islands bioinformatically predicted using the following criteria: (1) genomic area with ≥500 bp; (2) a percentage of GC ≥55 and; (3) the observed/expected CpG dinucleotides ≥0.65 [32]. Using the Ensembl database version 81 (GRCh38.p3)^1^, the web tool “CpG Island Searcher”^29^ and the described criteria, a single CpG island was predicted within/in the vicinity of murine *Dies1* locus at chr10:60.346.610-60.347.412. Concerning human *Dies1*, the same strategy was used and the predicted CpG island was located at chr10:71.773.335-71.773.735 (antisense strand). Custom designed primers (Sigma-Aldrich) used for bisulfite-treated-DNA amplification of murine/human *Dies1* promoter *CpG* island regions were: murine forward primer: 5′-GGGAAGTGGTTGGTTGGATA-3′; murine reverse primer: 5′-CCCATCCTACCCCCTACACT-3′; human forward primer 1: 5′-GAGGTAGATTTATTTTTTAGGT-3′; human reverse primer 1: 5′-CTATCTTCTCCCAAC-3′; human forward primer 2: 5′-AGGTAGTTTTTTTATA-3′ and human reverse primer 2: 5′-CTATCTTCTCCCAACTT-3′ (Sigma-Aldrich). Bisulfite-PCR products were sequenced for methylation-status determination using the same custom-designed primers in a 3130 Genetic Analyzer (Applied Biosystems).

### Short-interference-RNA experiments

Short-interference-RNA experiments were performed using mouse Dies1 siGENOME-SMARTpool (100 nM for 72 hours, Thermo Fisher Scientific), ON-TARGET plus Non-targeting siRNA #4 (100 nM for 72 hours, Thermo Fisher Scientific) as non-targeting control and, Lipofectamine 2000 (Thermo Fisher Scientific) as transfection agent.

### BMP4 treatment

BMP-pathway stimulation was performed using rhBMP4 (100 ng/mL for 48 hours, Immunotools).

### AntiMir experiments

AntiMiR experiments were performed using anti-miR miRNA inhibitor for hsa-miR-125a-5p or anti-miR Negative Control #1 (50 nM for 24 hours, Ambion) and Lipofectamine 3000 (Thermo Fisher Scientific) as transfection agent.

### Data mining of GC series

Microarray data from GC and gastrointestinal stromal tumours (GIST) human samples (GC series 3) collected from *ArrayExpress*^34^, ID: E-GEOD-26942, for probes ILMN_2205963 (*Dies1*), ILMN_1793990 (*ID2*) and ILMN_1732296 (*ID3*). Microarray data from normal myofibroblasts, adjacent myofibroblasts and cancer-associated gastric myofibroblasts collected from *ArrayExpress*, ID: E-GEOD-44740 for probes 225372_at (*Dies1*), 213931_at (*ID2*) and 207826_s_at (*ID3*)^35^. Analysed data from both experiments was used for boxplot representation and statistical significance assessed using Wilcoxon signed-rank test in R^51^.

## Additional Information

**How to cite this article**: Oliveira, P. *et al. Dies1/VISTA* expression loss is a recurrent event in gastric cancer due to epigenetic regulation. *Sci. Rep.* **6**, 34860; doi: 10.1038/srep34860 (2016).

## Supplementary Material

Supplementary Information
