# Supplementary material for: Dies1/VISTA expression loss is a recurrent event in gastric cancer due to epigenetic regulation
Source: Sci Rep. 2016 Oct 10;6:34860. doi: 10.1038/srep34860 (PMC5056517; doi:10.1038/srep34860)
Supplement: Supplementary Information [file srep34860-s1.pdf]

Supplementary Information

Manuscript Title:

Dies1/VISTA expression loss is a recurrent event in gastric cancer due to epigenetic regulation

List of Authors:

Patrícia Oliveira[1,2], Joana Carvalho[1,2], Sara Rocha[1,2], Mafalda Azevedo[2], Inês Reis[2], Vânia Camilo[2], Bárbara Sousa[1,2], Sofia Valente[1,2], Joana Paredes [1,2,3], Raquel Almeida[1,2,3], David Huntsman[4], Carla Oliveira[1,2,3*]

Affiliations:

[1] i3S - Instituto de Investigação e Inovação em Saúde, Universidade do Porto, Portugal

[2] IPATIMUP- Institute of Molecular Pathology and Immunology of the University of Porto (IPATIMUP), Rua Júlio Amaral de Carvalho,45, 4200-465 Porto, Portugal

[3]Dept. Pathology and Oncology, Faculty of Medicine, University of Porto, 4200-465 Porto, Portugal

[4]British Columbia Cancer Agency (BCCA), Vancouver V5Z 4E6, Canada

Supplementary Figure S1

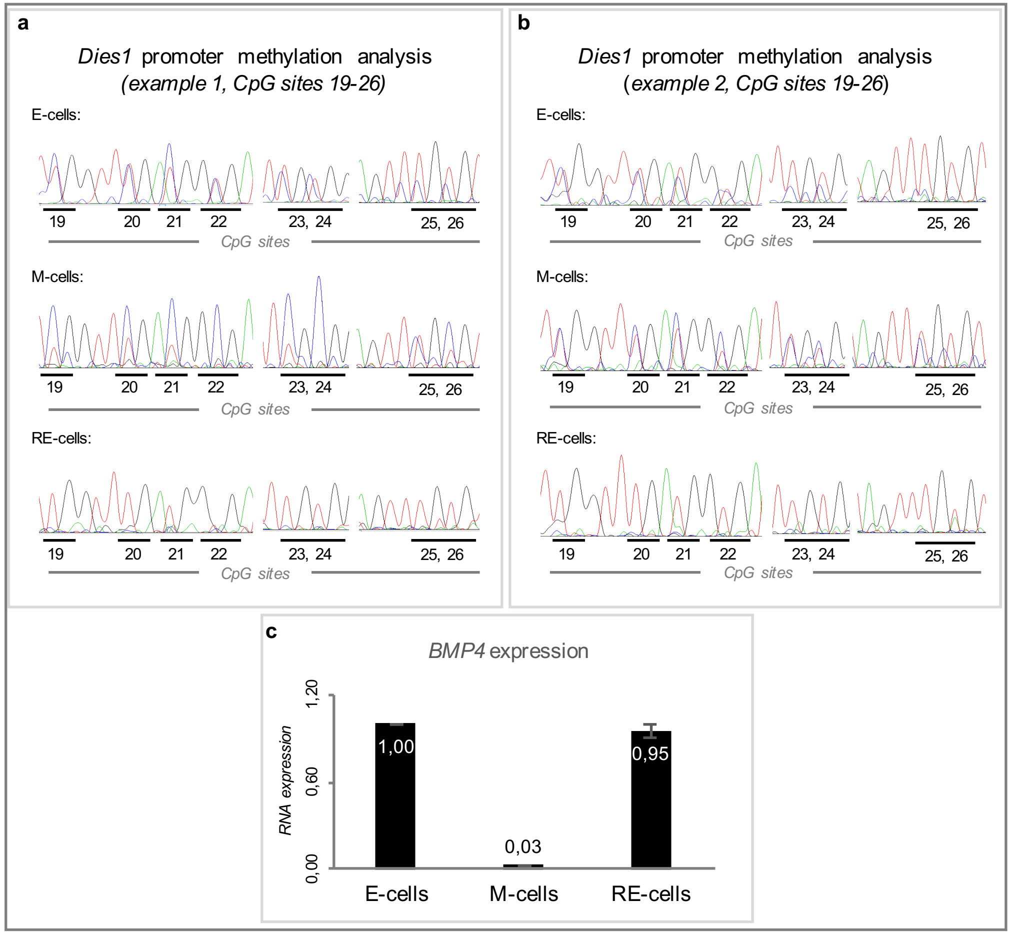

Supplementary Figure S1: *Dies1* promoter methylation. **a, b.** Electropherogram images for CpG sites 19-26, selected due to alterations in RE-cells, from two exemples of the results obtained for the direct-sequencing of *Dies1* predicted CpG island. **c.** *BMP4* RNA expression acros E-, M- and RE-cells. For M-cells, BMP4 is downregulated in concomitance with Dies1 expression.

Supplementary Figure S2

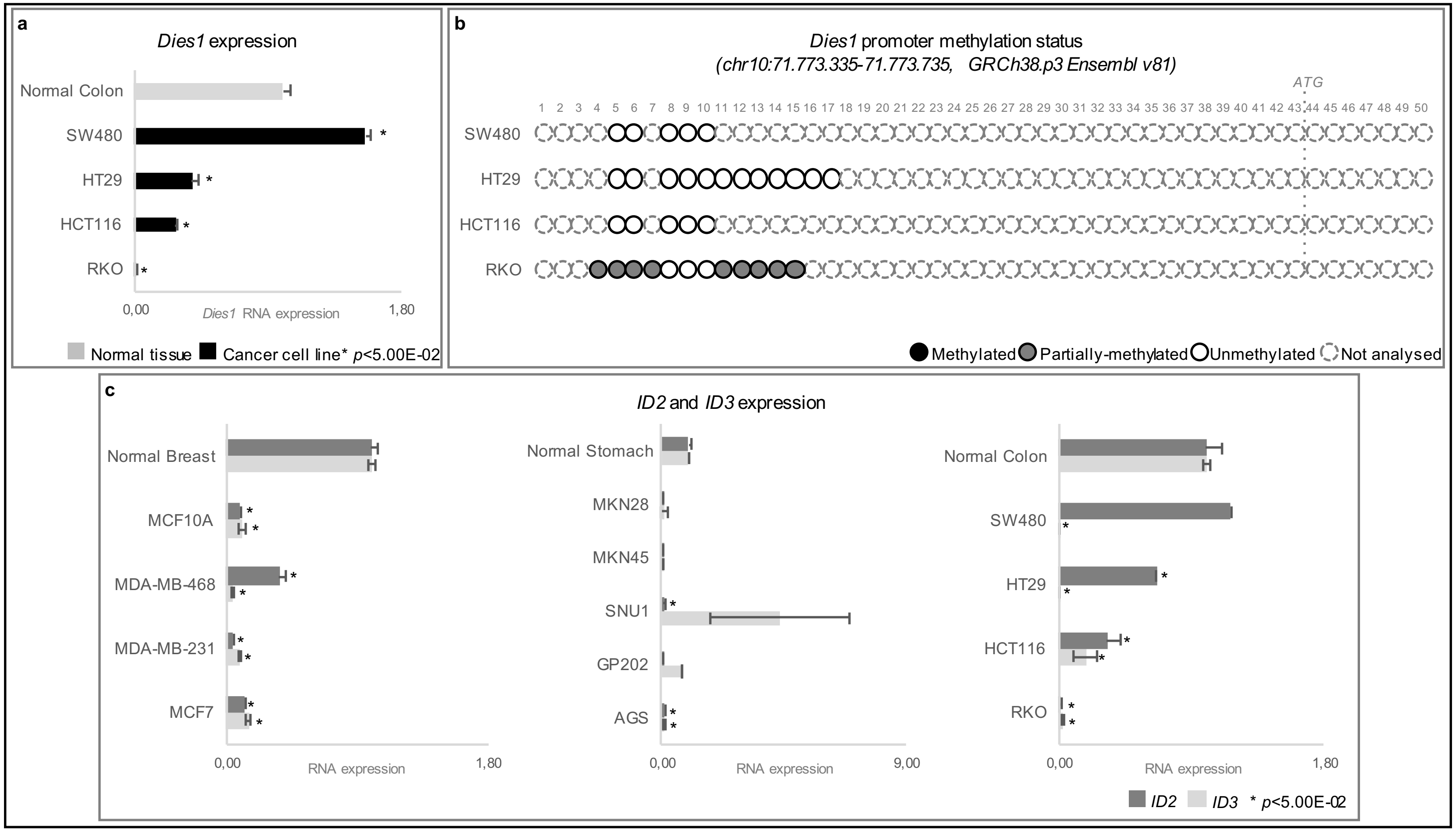

Supplementary Figure S2: *Dies1* Expression and promoter Methylation status in a panel of colorectal cancer cell lines. **A.** *Dies1* Expression in a panel of colorectal cancer cell lines (SW480, HT29, HCT116 and RKO, blackbars) normalized to normal colon tissue (light grey bar). Asterisks stand for significantly distinct comparisons (*p*<5.00E-02). **B.** *Dies1* promoter Methylation status in described colorectal cancer cell lines. Representation of each CpG site using white circles for unmethylated CpG sites, grey circles for partially-methylated CpG sites, black circles for fully methylated CpG sites and dashed white circles for unassessed CpG sites. **C.** *ID2* (grey) and *ID3* (light grey) Expression across the panel of breast, gastric and colorectal cancer cell lines. Expression data was normalized to the corresponding normal tissue. Non-cancer cell line MCF10A also assessed for *ID2* and *ID3* expression. Asterisks stand for significantly distinct comparisons (*p*<5.00E-02).

Supplementary Figure S3

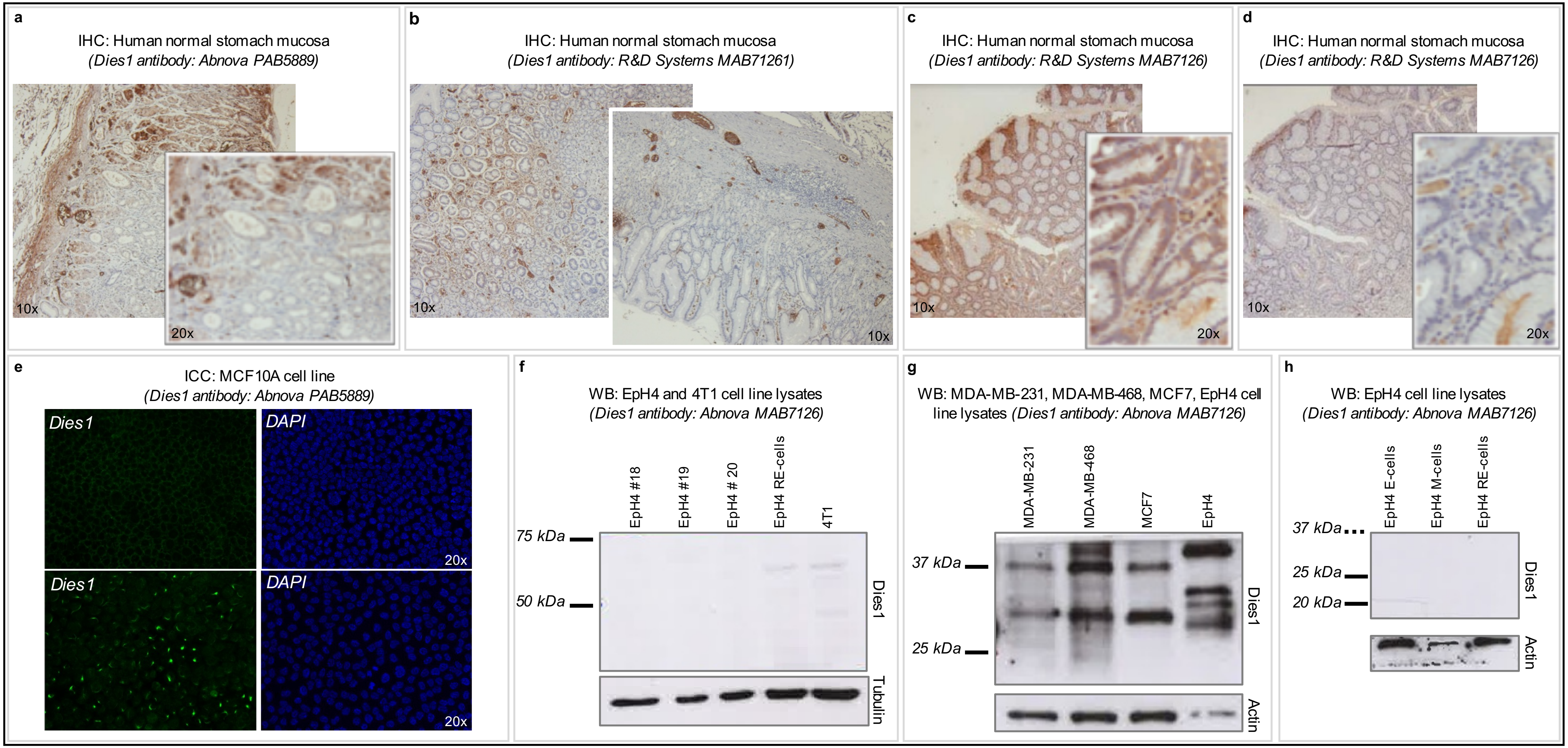

Supplementary Figure S3: Immunohistochemistry, immunocytochemistry and Western Blot results for Dies1 protein expression analysis using three anti-Dies1 antibodies. Antibodies used were PAB5889 (Abnova), MAB71261 (R&D Systems) and MAB7126 (R&D Systems), using manufacturer recommended conditions. **a-d.** Immunohistochemistry (IHC) of human normal stomach showing inconsistent and unreproducible Dies1 staining: **a.** different staining in the same histologic structure; **b.** different staining in the same histologic structure in the same slide; **c.** staining detected in some gastric glands and some stromal cells and; **d.** no staining detected in gastric glands and only some staining detected in stromal cells (same block section and protocol as in **c**). **e.** Immunocytochemistry (ICC) using Abnova PAB5889 anti-Dies1 antibody in MCF10A cells, again showing inconsistent results. Of notice, the same protocol was applied to different slides of MCF10A cells and different staining patterns were observed. **f-h.** Multiple tests using anti-Dies1 antibody MAB7126 for Western Blot (WB). **f.** WB for lysates from EpH4 cell line (different passages), EpH4 RE-cells and the murine breast cancer cell line 4T1. **g.** WB for lysates from MDA-MB-231, MDA-MB-468, MCF7 and EpH4 cells. **h.** WB for lysates from EpH4 E-, M- and RE-cells.

Supplementary Figure S4

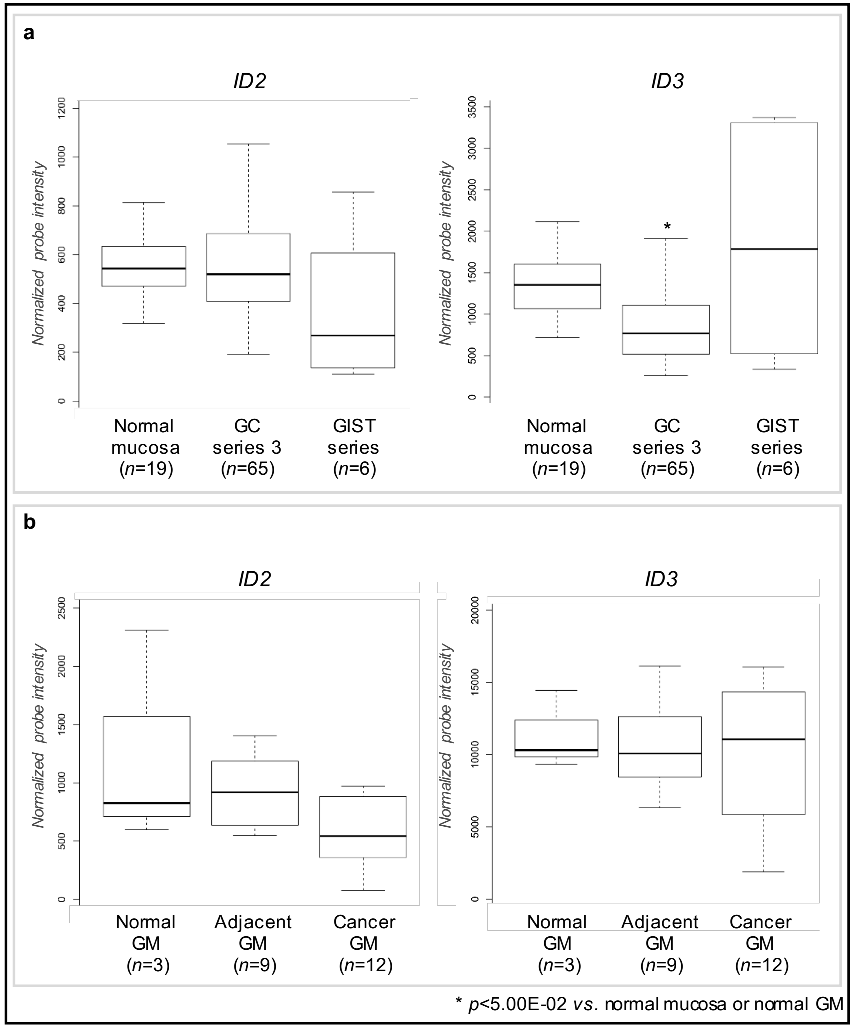

Supplementary Figure S4: *ID2* and *ID3* expression in GC Series 3 and in normal, adjacent and cancer-associated gastric myofibroblasts. Asterisks stand for significantly distinct comparisons (p<5.00E-02).
